# Pan-cancer analysis of TCGA data reveals notable signaling pathways

**DOI:** 10.1186/s12885-015-1484-6

**Published:** 2015-07-14

**Authors:** Richard Neapolitan, Curt M. Horvath, Xia Jiang

**Affiliations:** 1 Department of Preventive Medicine, Northwestern University Feinberg School of Medicine, Chicago, Il USA; 2 Department of Molecular Biosciences, Northwestern University, Evanston, Illinois USA; 3 Department of Biomedical Informatics, University of Pittsburgh, Pittsburgh, PA USA

**Keywords:** Pan-cancer, Breast cancer, Colon adenocarcinoma, Glioblastoma, Kidney renal papillary cell carcinoma, Low grade glioma, Lung adenocarcinoma, Lung squamous cell carcinoma, Ovarian carcinoma, Rectum adenocarcinoma, Uterine corpus endometriod carcinoma, Signal transduction pathway, Gene expression data, TCGA, SPIA

## Abstract

**Background:**

A *signal transduction pathway (STP)* is a network of intercellular information flow initiated when extracellular signaling molecules bind to cell-surface receptors. Many aberrant STPs have been associated with various cancers. To develop optimal treatments for cancer patients, it is important to discover which STPs are implicated in a cancer or cancer-subtype. The *Cancer Genome Atlas* (*TCGA*) makes available gene expression level data on cases and controls in ten different types of cancer including breast cancer, colon adenocarcinoma, glioblastoma, kidney renal papillary cell carcinoma, low grade glioma, lung adenocarcinoma, lung squamous cell carcinoma, ovarian carcinoma, rectum adenocarcinoma, and uterine corpus endometriod carcinoma. *Signaling Pathway Impact Analysis* (*SPIA*) is a software package that analyzes gene expression data to identify whether a pathway is relevant in a given condition.

**Methods:**

We present the results of a study that uses SPIA to investigate all 157 signaling pathways in the KEGG PATHWAY database. We analyzed each of the ten cancer types mentioned above separately, and we perform a pan-cancer analysis by grouping the data for all the cancer types.

**Results:**

In each analysis several pathways were found to be markedly more significant than all the other pathways. We call them notable. Research has already established a connection between many of these pathways and the corresponding cancer type. However, some of our discovered pathways appear to be new findings. Altogether there were 37 notable findings in the separate analyses, 26 of them occurred in 7 pathways. These 7 pathways included the 4 notable pathways discovered in the pan-cancer analysis. So, our results suggest that these 7 pathways account for much of the mechanisms of cancer. Furthermore, by looking at the overlap among pathways, we identified possible regions on the pathways where the aberrant activity is occurring.

**Conclusions:**

We obtained 37 notable findings concerning 18 pathways. Some of them appear to be new discoveries. Furthermore, we identified regions on pathways where the aberrant activity might be occurring. We conclude that our results will prove to be valuable to cancer researchers because they provide many opportunities for laboratory and clinical follow-up studies.

**Electronic supplementary material:**

The online version of this article (doi:10.1186/s12885-015-1484-6) contains supplementary material, which is available to authorized users.

## Background

A *signal transduction pathway (STP)* is a network of intercellular information flow initiated when extracellular signaling molecules bind to cell-surface receptors. The signaling molecules become modified, causing a change in their functional capability, affecting a change in the subsequent molecules in the network. This cascading process culminates in a cellular response. Consensus pathways have been developed based on the composite of studies concerning individual pathway components. KEGG PATHWAY [1] is a collection of manually drawn pathways representing our knowledge of the molecular interaction and reactions for about 157 signaling pathways. Signaling pathways are not stand-alone, but rather it is believed there is inter-pathway communication [2].

Many aberrant STPs have been associated with various cancers [3–9]. To develop optimal treatments for cancer patients, it is important to discover which STPs are implicated in a cancer or cancer-subtype. Microarray technology is providing us with increasingly abundant gene expression level datasets. For example, The *Cancer Genome Atlas* (*TCGA*) makes available gene expression level data on tumors and normal tissue in ten different types of cancer including breast cancer, colon adenocarcinoma, glioblastoma, kidney renal papillary cell carcinoma, low grade glioma, lung adenocarcinoma, lung squamous cell carcinoma, ovarian carcinoma, rectum adenocarcinoma, and uterine corpus endometriod carcinoma. Translating the information in these data into a better understanding of underlying biological mechanisms is of paramount importance to identifying therapeutic targets for cancer. In particular, if the data can inform us as to whether and how a signal transduction pathway is altered in the cancer, we can investigate targets on that pathway.

In an effort to reveal pathways implicated using gene expression data from tumors and normal tissue, researchers initially developed techniques such as over-representation analysis [10–12]. However these techniques analyze each gene separately rather than perform an analysis of the pathway at a systems level. By ignoring the topology of the network, they do not account for key biological information. That is, if a pathway is activated through a single receptor and that protein is not produced, the pathway will be severely impacted. However, a protein that appears downstream may have a limited effect on the pathway. Recently, researchers have developed methods that account for the topology.

*Signaling Pathway Impact Analysis* (*SPIA*) [13] is a software package (http://www.bioconductor.org/packages/release/bioc/html/SPIA.html) that analyzes gene expression data to identify whether a signaling network is relevant in a given condition by combining over-representation analysis with a measurement of the perturbation measured in a pathway. Neapolitan et al. [14] developed a method called *Causal Analysis of STP Aberrations* (*CASA*) for analysing signal pathways which represents signal pathways as causal Bayesian networks [15], and which also accounts for the topology of the network.

Even though much effort has been put into the development of these techniques for analyzing signaling pathways using gene expression data, it was not clear that we could get reliable results concerning signaling pathways by analyzing such data. That is, phosphorylation activity state of each protein in signaling pathway corresponds to the information flow on the pathway. Protein expression level (abundance) is correlated with activity, and gene expression level (mRNA abundance) is associated with protein abundance (correlation coefficient of 0.4 to 0.6). So, it seems gene expression data would be only loosely correlated with activity.

To investigate this question of whether we could obtain meaningful results using large-scale gene expression data, Neapolitan et al. [14] analyzed the ovarian cancer TCGA data using both SPIA and CASA. In their analysis, they investigated 20 signaling pathways believed to be implicated in cancer and 6 randomly chosen pathways. They obtained significant results that the cancers believed to be implicated in cancer are the ones most likely to be implicated in ovarian carcinoma.

The study in [14] was only a proof of principle study. In this paper we present the results of a study that uses SPIA to investigate all 157 signaling pathways in the KEGG PATHWAY database.

## Results and discussion

We analyzed all 157 signaling pathways in the KEGG PATHWAY database using SPIA. We performed a pan-cancer analysis that had all 2100 tumors, a breast cancer analysis that had 466 tumors, a colon adenocarcinoma analysis that had 143 tumors, a glioblastoma analysis that had 567 tumors, a kidney renal papillary cell carcinoma analysis that had 16 tumors, a low grade glioma analysis that had 27 tumors, a lung adenocarcinoma analysis that had 32 tumors, a lung squamous cancer analysis that had 154 tumors, an ovarian cancer analysis that had 572 tumors, a rectum adenocarcinoma analysis that had 69 tumors, and a uterine corpus endometriod carcinoma analysis that had 54 tumors. For all the analyses, we grouped the normal tissue samples from all the datasets, making a total of 101 normal tissue samples.

In all our analyses several pathways were found to be markedly more significant than the others, and also have very small FDRs. We call a pathway *notable* if the p-value is less than 0.0001 and the FDR is less than 0.01. We call a pathway *significant* if the p-value is less than 0.05. Table 1 shows the pathways found to be notable in all 11 of our analyses, and the most significant pathway that was not notable. Additional file 1: Tables S1-S11 show all pathways found to be significant (p-value < 0.05) in each of the analyses.

**Table 1:** The pathways found to be notable in the various analyses, and the most significant pathway that was not notable (listed last). A pathway is notable if the p-value is less than 0.0001 and the FDR is less than 0.01. A pathway is significant if the p-value is less than 0.05. The Status column gives the direction in which the pathway is found to be perturbed (activated or inhibited). The Signfct column contains an entry if the pathway is significant in the pan-cancer analysis. The entry is “N” if it is one of the notable pathways. Otherwise, it is “S”. A pathway has an asterisk if it is not notable in the pan-cancer analysis and previous studies have not linked it to the particular cancer

Analysis	Pathway	p-value	FDR	Status	Signfct
pan-cancer	Focal adhesion	5.99E-06	0.000789	Activated	N
	PI3K-Akt signaling pathway	1.01E-05	0.000789	Activated	N
	Rap1 signaling pathway	3.71E-05	0.001939	Activated	N
	Calcium signaling pathway	4.95E-05	0.001942	Activated	N
	Systemic lupus erythematosus	0.001966	0.05302	Activated	S
breast	ECM-receptor interaction	5.71E-05	0.008967	Activated	
	Complement and coagulation cascades	0.003855	0.218606	Activated	S
colon	Adrenergic signaling in cardiomyocytes*	3.35E-05	0.001709	Inhibited	S
	Melanoma	3.68E-05	0.001709	Inhibited	S
	Focal adhesion	4.73E-05	0.001709	Inhibited	N
	Cytokine-cytokine receptor interaction	5.84E-05	0.001709	Activated	S
	Pathways in cancer*	6.21E-05	0.001709	Inhibited	S
	PI3K-Akt signaling pathway	6.53E-05	0.001709	Inhibited	N
	Rap1 signaling pathway	0.002919	0.065477	Inhibited	N
glioblastoma	Cytokine-cytokine receptor interaction	5.12E-07	8.04E-05	Inhibited	S
	Complement and coagulation cascades*	1.33E-05	0.000798	Inhibited	S
	Systemic lupus erythematosus	1.94E-05	0.000798	Inhibited	S
	PI3K-Akt signaling pathway	2.31E-05	0.000798	Inhibited	N
	Chemokine signaling pathway	2.54E-05	0.000798	Inhibited	S
	Vascular smooth muscle contraction	0.003076	0.069809	Inhibited	
kidney	Rap1 signaling pathway	3.30E-06	0.000518	Inhibited	N
	ECM-receptor interaction*	8.13E-06	0.000638	Inhibited	
	Colorectal cancer*	2.79E-05	0.001459	Inhibited	
	Focal adhesion	8.66E-05	0.0034	Inhibited	N
	Insulin signaling pathway	0.000557	0.015232	Inhibited	
glioma	Focal adhesion	4.94E-06	0.000674	Inhibited	T
	ECM-receptor interaction*	8.59E-06	0.000674	Inhibited	
	Chemokine signaling pathway	1.74E-05	0.00091	Inhibited	S
	Small cell lung cancer*	4.27E-05	0.001482	Inhibited	S
	Cytokine-cytokine receptor interaction	4.72E-05	0.001482	Inhibited	S
	Retrograde endocannabinoid signaling	0.000478	0.01252	Activated	
**Analysis**	**Pathway**	**p-value**	**FDR**	**Status**	**Signfct**
lung adeno.	Chemokine signaling pathway	1.82E-08	2.86E-06	Activated	S
	Cytokine-cytokine receptor interaction	1.51E-05	0.001187	Activated	S
	Systemic lupus erythematosus	0.000108	0.005654	Activated	S
lung squamous	Chemokine signaling pathway	1.43E-05	0.002204	Activated	S
	Cytokine-cytokine receptor interaction	4.14E-05	0.002204	Activated	S
	Endocrine and other factor-reg. calcium reab.*	4.21E-05	0.002204	Inhibited	
	Amoebiasis	0.005649	0.221723	Inhibited	S
ovarian	Rap1 signaling pathway	4.02E-05	0.002785	Inhibited	N
	PI3K-Akt signaling pathway	5.03E-05	0.002785	Inhibited	N
	Calcium signaling pathway	5.32E-05	0.002785	Inhibited	N
	Focal adhesion	0.000366	0.014354	Inhibited	N
rectum	Focal adhesion	3.63E-06	0.000342	Inhibited	N
	Rap1 signaling pathway	4.36E-06	0.000342	Inhibited	N
	Ras signaling pathway*	1.32E-05	0.000689	Inhibited	S
	PI3K-Akt signaling pathway	4.96E-05	0.001727	Inhibited	N
	Prostate cancer*	5.50E-05	0.001727	Inhibited	S
	Melanoma	0.001514	0.039609	Inhibited	S
uterine	Focal adhesion	7.50E-07	0.000118	Inhibited	N
	Maturity onset diabetes of the young	4.69E-05	0.003144	Activated	S
	Calcium signaling pathway	6.01E-05	0.003144	Inhibited	N
	Rap1 signaling pathway	0.005318	0.208728	Inhibited	N

### Pan-cancer results

Table 1 reveals that the notable pathways in the pan-cancer analysis are the focal adhesion pathway, P13k-Akt pathway, Rap1 pathway, and calcium signaling pathways. This result verifies previous research showing that three of these four pathways are major players in cancer. The focal adhesion pathway has been shown to be involved in invasion, metastasis, angiogenesis, *epithelial-mesenchymal transition* (*EMT*), maintenance of cancer stem cells, and globally promoting tumor cell survival [16]. Furthermore, the *Focal Adhesion Kinase* (*FAK*) gene is a non-receptor tyrosine kinase that controls cellular processes such as proliferation, adhesion, spreading, motility, and survival [17–22]. FAK has been shown to be over-expressed in many types of tumors [23–26]. Disruption of FAK and p53 interaction with small molecule compound R2 reactivated p53 and blocked tumor growth [27]. The PI3K-Akt signaling pathway has been shown to be the most frequently altered pathway in human tumors. It controls most hallmarks of cancer, including cell cycle, survival, metabolism, motility and genomic instability; angiogenesis and inflammatory cell recruitment [28]. The Calcium signaling pathway has diverse functions in cellular regulation, which was found previously (with cell adhesion) by pathway analysis in breast cancer [29]. Yang et al. [30] discuss regulation of calcium signaling in lung cancer. On the other hand, much less is known about the Rap1 signaling pathway and cancer. There are only 6 pubmed citations concerning Rap1 and cancer. In particular, Bailey et al. [31] provide evidence to support a role for aberrant Rap1 activation in prostate cancer progression. Our results indicate Rap1 might be as big of a player in all cancers as the other three pathways just discussed.

### Individual cancer results

Next we discuss the individual cancer results. Each of these discussions refers to information provided in Table 1.

The only notable pathway in the breast cancer analysis is the ECM-receptor interaction pathway. This pathway was not found to be significant in the pan-cancer analysis, much less notable. However, previous research links changes in the extracellular matrix (ECM) to breast cancer. Lu et al. [32] recently discuss how the ECM’s biomechanical properties change under disease conditions. In particular, tumor stroma is typically stiffer than normal stroma; and in the case of breast cancer, diseased tissue can be 10 times stiffer than normal breast tissue.

There are 7 notable pathways in the case of colon adenocarcinoma, and all of them were found to be significant in the pan-cancer analysis. The PI3k-Akt signaling pathway and focal adhesion pathway were both found to be notable in the pan-cancer analysis and were discussed above. There are only 7 pubmed citations linking the highest ranking pathway, adrenergic signaling in cardiomyocytes, to cancer. The second pathway, namely the melanoma pathway, is of course linked to cancer. Furthermore, there is research substantiating that the BRAF mutation is prominent in melanoma and colorectal cancer [33]. BRAF is on the melanoma pathway. As to the cytokine-cytokine receptor interaction pathway, there has been research linking cytokine receptors to colorectal cancer [34]. The pathway in cancer pathway is of course linked to cancer. Our result substantiates its role in colon cancer in particular.

The top ranking pathway in the case of glioblastoma is the cytokine-cytokine receptor interaction pathway, whose relevance to cancer we just discussed. The second pathway is complement and coagulation cascades. Recent research has suggested an essential role of this pathway in multiple cancers [35], but not glioblastoma in particular. Our results support that it is also has a role in glioblastoma. The third pathway, namely system lupus erythematosus, has been linked to glioblastoma [36]. We have already discussed the PI3K-Akt signalling pathway, as it was one of the notable pathways in the pan-cancer analysis. Finally, chemokine signaling has been associated with a number of cancers including glioma [37].

The first and fourth pathways for kidney renal papillary cell carcinoma are two of the notable pathways in the pan-cancer analysis, and have already been discussed. The second pathway, namely the ECM-receptor interaction pathway was also discussed because it was the most significant pathway in breast cancer. Finally, the colorectal cancer pathway is of course linked to cancer, but we know of no specific study implicating it in kidney renal papillary cell carcinoma.

The chemokine signaling pathway and the cytokine-cytokine receptor interaction pathway are both notable in low grade glioma. These same two pathways were found to be significant in glioblastoma and were discussed above. The first pathway, namely focal adhesion, is one of the notable pathways in our pan-cancer analysis. The second pathway, ECM-receptor interaction, was previously discussed because it was the most notable pathway in breast cancer. Finally, the small cell lung cancer pathway is concerned with cancer, but a literature search did not reveal any study linking it specifically to glioma.

The two notable pathways in the case of lung adenocarcinoma are also notable in glioblastoma, and were discussed when we discussed that cancer. The cytokine-cytokine receptor interaction pathway has been implicated specifically with lung cancer [38], as has chemokine signaling [39].

The top two pathways in the case of lung squamous cell carcinoma are the same as the top two in the case of lung adenocarcinoma. Their relevance to lung cancer was just discussed. A pubmed search does not show any papers linking cancer with the third pathway, endocrine and other factor-regulated calcium absorption.

The notable pathways in ovarian cancer are all notable pathways in the pan-cancer analysis, and were previously discussed.

Three of the notable pathways in the rectum adenocarcinoma analysis, are notable pathways in the pan-cancer analysis. The third ranked pathway, RAS signaling, has been associated with renal carcinoma [40]. As to the prostate cancer pathway, prostate cancer and renal cell cancer have been shown to have some commonality [41].

Two of the three notable pathways for uterine corpus endometriod carcinoma are notable pathways in the pan-cancer analysis. As to the third pathway, the connection between maturity onset diabetes of the young and endometrial cancer has been well-established [42].

### Summary results

Out of 157 signaling pathways analyzed, only 18 were found to be notable in at least one cancer. Table 2 lists those pathways. Out of a total of 37 notable findings, 26 occurred for the top 7 pathways. So, our results indicate that relatively few pathways are responsible for much of the aberrant activity in cancer. Of those 7 pathways, 4 were found to be notable in the pan-cancer analysis, and 2 others were fairly significant (p-values of 0.006 and 0.007). So these pathways may play roles in many different cancers. However, the ECM-receptor interaction pathway was not significant in the pan-cancer analysis (p-value of 0.472), indicating that perhaps this pathway is relevant only to the 3 cancers in which it was found to be notable, namely breast cancer, kidney renal papillary cell carcinoma, and low grade glioma.

**Table 2:** The pathways that were found to be notable in at least one cancer analysis. The second column shows the number of cancer types in which the pathway was found to be notable. The pathways are ranked by that column. The third column contains an “N” if the pathway was found to be notable in the pan-cancer analysis and it contains an “S” if it was only found to be significant in the pan-cancer analysis. The fourth column shows the p-value in the pan-cancer analysis

Rank	Pathway	# cancers	Pan_cancer	p-value
1	Focal Adhesion	5	N	5.99E-06
2	Cytokine-cytokine receptor interaction	5	S	0.006
3	PI3K-Akt signaling pathway	4	N	1.01E-05
4	Chemokine signaling pathway	4	S	0.007
5	Rap1 signaling pathway	3	N	3.71E-05
6	ECM-receptor interaction	3		0.472
7	Calcium signaling pathway	2	N	4.95E-05
8	Adrenergic signaling in cardiomyocytes	1	S	0.014
9	Melanoma	1	S	3.00E-03
10	Pathways in Cancer	1	S	0.002
11	Complement and coagulation cascades	1	S	0.005
12	Systemic lupus erythematosus	1	S	0.002
13	Colerectal cancer	1		0.531
14	Small cell lung cancer	1	S	0.015
15	Endocrine and other factor-regulated calcium reabsorption	1		0.183
16	Ras signal pathway	1	S	0.038
17	Prostate cancer	1	S	0.004
18	Maturity onset diabetes of the young	1	S	0.047

To gain insight as to how much each particular cancer has in common with all cancers, we computed the Jaccard Index comparing the notable pathways in the each cancer type to the notable pathways in the pan-cancer analysis. If *A* and *B* are the two sets, the Jaccard Index of *A* and *B* is given by

$$ J\left(A,B\right)=\frac{\left|A\cap B\right|}{\left|A\cup B\right|}, $$

where *A* is the number of items in *A*. The value of *J*(*A*, *B*) is 0 if *A* and *B* have no items in common, and is 1 if *A* and *B* are the same set.

Table 3 shows the Jaccard Indices. Ovarian carcinoma is at the top with an index of 0.75. The index would have been even higher, namely 1.0, if we had included the fourth most significant pathway for Ovarian Cancer, which is Focal adhesion and has a p-value of 0.000366. At the bottom we have breast cancer and the two lung cancers with Jaccard Indices equal to 0.

**Table 3:** The Jaccard Index for each cancer type. The index is based on the number of notable pathways the cancer analysis has in common with the pan-cancer analysis

Cancer type	Jaccard index
Ovarian carcinoma	0.75
Rectum adenocarcinoma	0.6
Uterine corpus Endometriod carcinoma	0.4
Kidney renal papillary cell carcinoma	0.333
Colon adenocarcinoma	0.222
Glioblastoma	0.125
Low grade glioma	0.125
Breast cancer	0
Lung adenocarcinoma	0
lung squamous cell carcinoma	0

### Pathway intersections

**Fig. 1:**
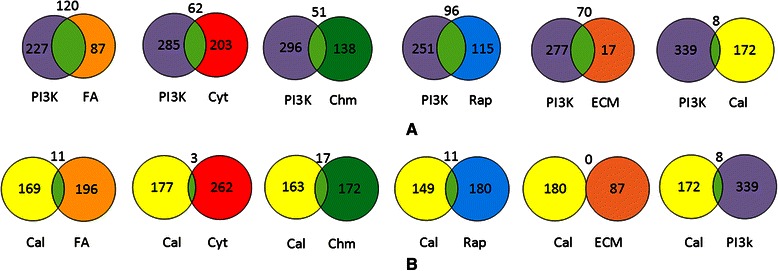
Venn diagrams showing number of proteins pathway pairs have in common. **a**) Intersection of PI3K-Akt with each of the other top 6 pathways. **b**) Intersection of calcium signalling pathway with each of the other top 6 pathways

If we look at the pathway diagrams for our seven most significant pathways appearing in Table 2, often different signaling molecules bind to different receptors (integrin, RTK, GPCR), but the responses converge on many of the same proteins. For example, PI3K-Akt, Focal Adhesion, and Rap1 all converge on protein PI3K. To gain insight as to how much overlap there is among the seven most significant pathways, we determined the number of proteins each pathway pair has in common. The results appear in Table 4. Two interesting relationships are discernable in that table, and they are depicted in Fig. 1.

**Table 4:** The number of proteins that the top 7 pathways have in common with each other. The entry is the number of proteins that are affiliated with both of the two indicated pathways

	FA	Cyt	PI3k	Chm	Rap	ECM	Cal
**FA**	207	16	120	44	63	70	11
**Cyt**	16	265	62	64	21	0	3
**PI3K**	120	62	347	51	96	70	8
**Chm**	44	64	51	189	51	0	17
**Rap**	63	21	96	51	211	4	31
**ECM**	70	0	70	0	4	87	0
**Cal**	11	3	8	17	31	0	180

The first relationship is that PI3K-Akt has substantial overlap will five of the other six pathways. This is shown in Fig. 1a. PI3K-Akt is “probably one of the most important pathways in cancer metabolism and growth” [43]. The fact that it overlaps substantially will five other significant pathways indicates that much of the aberrant signaling in many cancers might be located in regions where PI3K-Akt overlaps with other pathways.

The second interesting relationship is that the Calcium pathway hardly overlaps with the other six pathways. This is shown in Fig. 1b. The Calcium pathway was found to be notable in only ovarian and uterine cancer (Table 1). This result indicates that there might be a common region of aberrant signaling in these two cancers, which does not overlap with regions of aberrant signaling in other cancers.

**Fig. 2:**
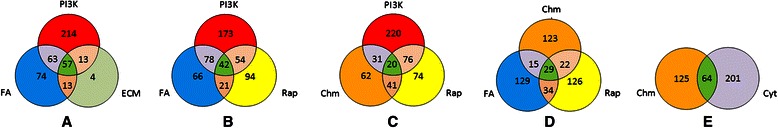
Venn diagrams showing number proteins pathway triplets have in common. **a**) PI3K-Akt, focal adhesion, and Rap1. **b**) P13K-Akt, focal adhesion, and Rap1. **c**) P13K-Akt, chemokine signaling, and Rap1. **d**) chemokine signaling, focal adhesion, and Rap1. **e**) chemokine signaling, and cytokine-cytokine receptor interaction. In each of the diagrams, the intersection of the pathways includes essentially no proteins from the other significant pathways

To discover possible hotspots where other aberrant signaling might occur, we looked at higher order intersections. We discovered the intersections shown in Fig. 2. In each of the diagrams in that figure, the intersection of the pathways in the diagram includes essentially no proteins from the other significant pathways.

Perhaps the most interesting relationship appears in Fig. 2a, which shows that the majority of the proteins in the ECM-receptor interaction pathway are located in the intersection of the PI3K-Akt and Focal Adhesion pathways. The ECM-receptor interaction pathway was found to be notable in breast cancer, kidney cancer, and glioma. This result indicates that there may be a region of aberrant signaling, located in the intersection of PI3K-Akt and Focal Adhesion, in these cancers.

Figures 2b and c show other possible hot regions in PI3K-Akt, while Fig. 2d and e show possible hot regions not including PI3K-Akt. Of these figures, Fig. 2e is the most compelling. The Cytokine-cytokine receptor interaction and Chemokine signaling pathways have a large intersection that excludes other pathways. Both these pathways were found to be notable in glioblastoma, glioma, lung adenocarcinoma, and lung squamous cancer. Only the Cytokine-cytokine receptor interaction pathway was found to be notable in colon cancer. So there may be a region of aberrant signaling, located in the intersection of these pathways, in these cancers.

### Cancer clusters

**Fig. 3:**
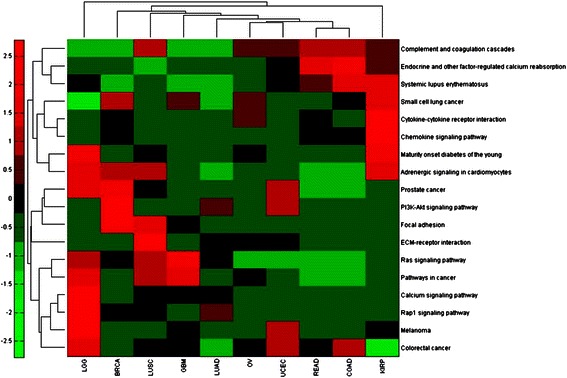
Heat map showing cancer and pathway clusters. The entries are standardized values of the p-value. The p-values are mapped to [−0.5, 0.5]; then standardization is done along the rows by the hierarchical clustering algorithm in MATLAB so that the mean values is 0 and the standard deviation is 1. Abbreviations: LGG: low grade glioma; BRCA: breast; LUSC: lung squamous; GBM: glioblastoma; LUAD: lung adenocarcinoma; OV: ovarian; UCEC: uterine; READ: rectum; COAD: colon; KIRP: kidney

To investigate further how different cancers might share common causal mechanisms, we developed a heat map, based on hierarchical clustering, with cancer type on the horizontal, the 18 notable pathways on the vertical, and with the entry being p-value. Figure 3 shows the heat map. Ovarian cancer and uterine cancer constitute a primary group. This is consistent with our result mentioned about that the calcium pathway was found to be notable only in these two cancers. Furthermore, these cancers are in close proximity. Rectum cancer and colon cancer also constitute a primary group, which is consistent with their close proximity.

## Discussion

We performed a pan-cancer analysis by grouping the TCGA data on 10 different cancer types. We identified 4 signaling pathways to be markedly more significant (which we called notable) than the remaining 153 pathways. We also did a separate analysis for each of the 10 types of cancers individually. In all 10 of the cancers, there were several pathways that were found to be markedly more significant than the others. Altogether there were 37 notable findings in the separate analyses, and 26 of them occurred in 7 pathways. These 7 pathways included the 4 discovered in the pan-cancer analysis. Our results suggest that these 7 pathways account for much of the mechanisms of cancer.

As we discussed, research has already established a connection between many of the 18 pathway we discovered and the corresponding cancer type. However, some of them appear to be new discoveries. Furthermore, we have identified regions on the pathways that might account for the aberrant behaviour. So, we have both substantiated previous knowledge, and provided researchers with avenues for future investigations.

The PI3K-Akt pathway has long been recognized as an aberrant pathway in breast cancer [43]. However, our breast cancer analysis did not find it to be significant (p = 0.304). On the other hand, the ECM-receptor interaction pathway was the only notable pathway in the breast cancer analysis, and we showed that 70 of its 87 proteins are on the PI3K-Akt pathway. So, our results indicate that the effect of PI3K-Akt on breast cancer might be localized in this region of the PI3K-Akt pathway.

It likely that there are other known pathways that affect various cancers, which we did not discover. The analysis of gene expression alone may not account for pathways that are activated by post-translational modification (like phosphorylation/dephos) that could change the pathway activation profile without altering mRNA abundance. So, we should interpret our results only as suggesting avenues of investigation, rather than as disconfirming any existing knowledge.

This in silico analysis of cancer patient signaling pathways provides many opportunities for laboratory and clinical follow-up studies. We know of no dataset as comprehensive as the TCGA datasets. However, there are individual datasets for specific cancers that could be investigated. For example, the Molecular Taxonomy of Breast Cancer International Consortium (METABRIC) dataset has data on 1981 breast cancer tumors, and expression levels for 16,384 genes [44].

## Conclusions

We presented the results of a study that analyzes all 157 signaling pathways in the KEGG PATHWAY database using TCGA gene expression datasets concerning ten types of cancer. We performed a pan-cancer analysis and analyze each dataset separately. There were 37 notable findings concerning 18 pathways. Research has already established a connection between many of these pathways and the corresponding cancer type. However, some of them appear to be new discoveries. Furthermore, we identified regions on pathways where the aberrant activity might be occurring. We conclude that our results will prove to be valuable to cancer researchers because they provide many opportunities for laboratory and clinical follow-up studies.

## Method

This research does not involve any human subjects. It utilizes the publically available de-identified TCGA datasets. *The Cancer Genome Atlas* (*TCGA*) makes available datasets concerning breast cancer, colon adenocarcinoma, glioblastoma, kidney renal papillary cell carcinoma, low grade glioma, lung adenocarcinoma, lung squamous cell carcinoma, ovarian carcinoma, rectum adenocarcinoma, and uterine corpus endometriod carcinoma. Each dataset contains data on the expression levels of 17,814 genes in tumorous tissue and in normal tissue. Table 5 shows the number of tumor samples and non-tumor samples in each of these datasets. Tables 6, 7, 8, 9, 10 shows demographic information concerning the patients from which the samples were taken.

**Table 5:** The number of tumor samples and normal samples in the TCGA cancer datasets

Cancer	# tumors	# normal
Breast cancer	466	61
Colon adenocarcinoma	143	19
Glioblastoma	567	10
Kidney renal papillary cell carcinoma	16	0
Low grade glioma	27	0
Lung adenocarcinoma	32	0
Lung squamous cell carcinoma	154	0
Ovarian carcinoma	572	8
Rectum adenocarcinoma	69	3
Uterine corpus endometriod carcinoma	54	0
Pan-cancer (total)	2100	101

**Table 6:** Gender distribution of the patients from which the various samples were obtained

Cancer	Tumor samples		Non-tumor samples	
	Female	Male	Female	Male
Breast cancer	461	5	60	1
Colon adenocarcinoma	67	76	14	5
Glioblastoma	219	348	5	5
Kidney renal papillary cell carcinoma	4	12	0	0
Low grade glioma	9	18	0	0
Lung adenocarcinoma	18	14	0	0
Lung squamous cell carcinoma	44	110	00	0
Ovarian carcinoma	572	0	8	0
Rectum adenocarcinoma	31	38	3	0
Uterine corpus endometriod carcinoma	54	0	0	0
Pan-cancer (total)	1479	621	90	11

**Table 7:** Menopause status distribution of the patients from which the various samples were obtained

Cancer	Tumor samples				Non-tumor samples			
	Pre	Peri	Post	NA	Pre	Peri	Post	NA
Breast cancer	104	16	297	49	19	2	28	12
Colon adenocarcinoma	0	0	0	143	0	0	0	19
Glioblastoma	0	0	0	567	0	0	0	10
Kidney renal papillary cell carcinoma	0	0	0	16	0	0	0	0
Low grade glioma	0	0	0	27	0	0	0	0
Lung adenocarcinoma	0	0	0	32	0	0	0	0
Lung squamous cell carcinoma	0	0	0	154	0	0	0	0
Ovarian carcinoma	0	0	0	572	0	0	0	8
Rectum adenocarcinoma	0	0	0	69	0	0	0	3
Uterine corpus endometriod carcinoma	5	0	45	4	0	0	0	0
Pan cancer (total)	109	16	342	1633	19	2	28	52

**Table 8:** Race distribution of the patients from which the various samples were obtained. Ind: American indian or Alaska native; Asn: Asian; Blk: Black or African American; Haw: Native Hawaiian or other Pacific islander; Wht: white; NA: Not available

Cancer	Tumor samples						Non-tumor samples					
	Ind.	Asn.	Blk.	Haw.	Wht.	NA	Ind.	Asn.	Blk.	Haw.	Wht.	NA
Breast cancer	1	34	39	0	303	89	0	0	1	0	59	1
Colon adenocarcinoma	0	0	1	0	9	133	0	0	2	0	8	9
Glioblastoma	0	13	34	0	495	25	0	0	0	0	0	10
Kidney renal papillary cell carcinoma	0	0	0	0	9	7	0	0	0	0	0	0
Low grade glioma	0	0	2	0	25	0	0	0	0	0	0	0
Lung adenocarcinoma	0	2	1	0	26	3	0	0	0	0	0	0
Lung squamous cell carcinoma	0	3	7	0	91	53	0	0	0	0	0	0
Ovarian carcinoma	3	19	24	1	493	32	0	0	0	0	0	8
Rectum adenocarcinoma	0	0	1	0	4	64	0	0	0	0	3	0
Uterine corpus endometriod carcinoma	2	4	6	0	40	2	0	0	0	0	0	0
Pan-cancer (total)	6	75	115	1	1495	408	0	0	3	0	70	28

**Table 9:** Ethnicity distribution of the patients from which the various samples were obtained

Cancer	Tumor samples			Non-tumor samples		
	Latino	Not Latino	NA	Latino	Not Latino	NA
Breast cancer	7	336	123	0	36	25
Colon adenocarcinoma	0	10	133	0	10	9
Glioblastoma	12	465	90	0	0	10
Kidney renal papillary cell carcinoma	0	16	0	0	0	0
Low grade glioma	1	20	6	0	0	0
Lung adenocarcinoma	1	28	3	0	0	0
Lung squamous cell carcinoma	4	88	62	0	0	0
Ovarian carcinoma	11	330	231	0	0	8
Rectum adenocarcinoma	0	5	64	0	3	0
Uterine corpus endometriod carcinoma	2	24	28	0	0	0
Pan-cancer (total)	13	1322	740	0	49	52

**Table 10:** Age distribution of the patients from which the various samples were obtained

Cancer	Tumor samples						Non-tumor samples					
	0-20	21-40	41-60	61-80	81-100	NA	0-20	21-40	41-60	61-80	81-100	NA
Breast cancer	0	51	198	194	22	1	0	7	26	25	3	0
Colon adenocarcinoma	0	2	22	90	29	0	0	0	3	12	4	0
Glioblastoma	7	63	238	237	20	2	0	1	4	4	1	0
Kidney renal papillary cell carcinoma	0	0	11	5	0	0	0	0	0	0	0	0
Low grade glioma	1	15	10	1	0	0	0	0	0	0	0	0
Lung adenocarcinoma	0	1	9	20	2	0	0	0	0	0	0	0
Lung squamous cell carcinoma	0	2	31	112	7	2	0	0	0	0	0	0
Ovarian carcinoma	0	23	295	233	20	1	0	4	4	0	0	0
Rectum adenocarcinoma	0	1	14	47	7	0	0	0	1	2	0	0
Uterine corpus endometriod carcinoma	0	3	23	22	6	0	0	0	0	0	0	0
Pan-cancer (total)	8	161	851	961	113	6	0	12	38	43	8	0

We did a pan-cancer analysis by grouping the ten different cancer datasets into one dataset, resulting in 2100 tumor samples and 101 normal samples.

*KEGG* (*Kyoto Encyclopedia of Genes and Genomes*) is a database resource that integrates genomic, chemical and systemic functional information. We chose KEGG because it is widely used as a reference knowledge base for integration and interpretation of large-scale datasets generated by genome sequencing and other high-throughput experimental technologies. KEGG PATHWAY [1] is a collection of manually drawn pathway maps representing our knowledge on the molecular interaction and reaction networks for the following:

1. Metabolism
  Global/overview, Carbohydrate, Energy, Lipid, Nucleotide, Amino acid,
  Other amino, Glycan, Cofactor/vitamin, Terpenoid/PK,
  Other secondary metabolite, Xenobiotics, Chemical structure
2. Genetic Information Processing
3. Environmental Information Processing
4. Cellular Processes
5. Organismal Systems
6. Human Diseases

We investigated all 157 signaling pathways in the KEGG databases. For each pathway, we identified all the genes related to the pathways. We extracted gene expression profiles for the 2100 tumor samples and 101 normal samples in the TCGA database. By mapping the gene names of the genes in the gene sets identified using KEGG pathways and the gene names in TCGA data, we were able to extract the gene expression profiles for each of the 157 pathways for the 2100 tumor samples and 101 normal samples. The TCGA gene expression data is already processed and normalized.

We repeated this procedure for each of the ten cancer datasets separately. Each dataset has the number of tumor samples shown in Table 5. However, to achieve a larger sample for the normal samples, we grouped the normal samples in the ten datasets, making the number of normal samples equal to 101.

Once these datasets were developed, we analysed each dataset using the software package SPIA [13] (http://www.bioconductor.org/packages/release/bioc/html/SPIA.html), which analyzes gene expression data to identify whether a signaling pathway is relevant in a given cancer by 1) determining the overrepresentation of genes on the pathway that are differentially expressed in tumor samples versus normal samples; and 2) investigating the abnormal perturbation of the pathway, as measured by propagating measured expression changes across the pathway topology. SPIA produces a p-value showing the significance level at which a pathway is found to be perturbed in cancerous tissue and a *false discovery rate* (*FDR*). We ran SPIA using the recommended value of 2000 bootstrap iterations, and all parameters set to their default values.

## Additional file

Additional file 1: **These 11 tables show all pathways found to be significant (p-value < 0.05) in each of the analyses.** **Table S1.** The pathways found to be significant in the pan-cancer analysis. **Table S2.** The pathways found to be significant in the breast cancer analysis. The far right column contains an entry if the pathway was found to be significant in the pan-cancer analysis. The entry is “H” if it was one of the highly significant pathways. Otherwise, it is “S”. **Table S3.** The pathways found to be significant in colon adenocarcinoma analysis. The far right column contains an entry if the pathway was found to be significant in the pan-cancer analysis. The entry is “H” if it was one of the highly significant pathways. Otherwise, it is “S”. **Table S4.** The pathways found to be significant in the glioblastoma analysis. The far right column contains an entry if the pathway was found to be significant in the pan-cancer analysis. The entry is “H” if it was one of the highly significant pathways. Otherwise, it is “S”. **Table S5.** The pathways found to be significant in the Kidney Renal Papillary Cell Carcinoma analysis. The far right column contains an entry if the pathway was found to be significant in the pan-cancer analysis. The entry is “H” if it was one of the highly significant pathways. Otherwise, it is “S”. **Table S6.** The pathways found to be significant in the Low Grade Glioma analysis. The far right column contains an entry if the pathway was found to be significant in the pan-cancer analysis. The entry is “H” if it was one of the highly significant pathways. Otherwise, it is “S”. **Table S7.** The pathways found to be significant in the Lung Adenocarcinoma analysis. The far right column contains an entry if the pathway was found to be significant in the pan-cancer analysis. The entry is “H” if it was one of the highly significant pathways. Otherwise, it is “S”. **Table S8.** The pathways found to be significant in the lung squamous cell carcinoma analysis. The far right column contains an entry if the pathway was found to be significant in the pan-cancer analysis. The entry is “H” if it was one of the highly significant pathways. Otherwise, it is “S”. **Table S9.** The pathways found to be significant in the ovarian cancer analysis. The far right column contains an entry if the pathway was found to be significant in the pan-cancer analysis. The entry is “H” if it was one of the highly significant pathways. Otherwise, it is “S”. **Table S10.** The pathways found to be significant in the rectum adenocarcinoma analysis. The far right column contains an entry if the pathway was found to be significant in the pan-cancer analysis. The entry is “H” if it was one of the highly significant pathways. Otherwise, it is “S”. **Table S11.** The pathways found to be significant in the uterine corpus endometrioid carcinoma analysis. The far right column contains an entry if the pathway was found to be significant in the pan-cancer analysis. The entry is “H” if it was one of the highly significant pathways. Otherwise, it is “S”.

## References

[1] KEGG PATHWAY: http://www.genome.jp/kegg/pathway.html.

[2] Ideker T, Galitski T, Hood L (2001). A new approach to decoding life: systems biology. Annu Rev Genomics Human Gen.

[3] Ciriello G, Cerami E, Sander C, Schultz N (2012). Mutual exclusivity analysis identifies oncogenic network modules. Genome Res.

[4] Vandin F, Upfal E, Raphael BJ: De novo discovery of mutated driver pathways in cancer. *Genome Research* 2011, 1–12: doi:10.1101/gr.120477.111. 10.1101/gr.120477.111 PMC3266044 21653252

[5] Vandin F, Upfal E, Raphael BJ (2011). Algorithms for detecting significantly mutated pathways in cancer. J Comput Biol.

[6] Zhao J, Zhang S, Wu L-Y, Zhang X-S (2012). Efficient methods for identifying mutated driver pathways in cancer. Bioinformatics.

[7] Jebar AH, Hurst CD, Tomlinson DC, Johnston C, Taylor CF, Knowles MA (2005). FGFR3 and Ras gene mutations are mutually exclusive genetic events in urothelial cell carcinoma. Oncogene.

[8] Kurose K (2002). Frequent somatic mutations in PTEN and TP53 are mutually exclusive in the stroma of breast carcinomas. Nat Genet.

[9] Xing M (2004). Early occurrence of RASSF1A hypermethylation and its mutual exclusion with BRAF mutation in thyroid tumorigenesis. Cancer Res.

[10] Drặghici S (2003). Global functional profiling of gene expression. Genomics.

[11] Subramanian A (2005). Gene set enrichment analysis: a knowledge-based approach for interpreting genome-wide expression profiles. Proc Natl Acad Sci U S A.

[12] Tian L (2005). Discovering statistically significant pathways in expression profiling studies. Proc Natl Acad Sci U S A.

[13] Tarca A (2009). A novel signaling pathway impact analysis. Bioinformatics.

[14] Neapolitan R, Jiang X (2014). Inferring aberrant signal transduction pathways in ovarian cancer from TCGA Data. Cancer Informat.

[15] Neapolitan RE (2003). Learning Bayesian Networks.

[16] Cance WG, Kurenova E, Marlowe T, Golubovskaya V (2013). Disrupting the scaffold to improve focal adhesion kinase-targeted cancer therapeutics. Sci Signal.

[17] Hanks SK, Polte TR (1997). Signaling through focal adhesion kinase. Bioessays.

[18] Mitra SK, Schlaepfer DD (2006). Integrin-regulated FAK-Src signaling in normal and cancer cells. Curr Opin Cell Biol.

[19] McLean GW (2005). The role of focal-adhesion kinase in cancer - a new therapeutic opportunity. Nat Rev Cancer.

[20] Schaller MD (2010). Cellular functions of FAK kinases: insight into molecular mechanisms and novel functions. J Cell Sci.

[21] Guan JL (1997). Role of focal adhesion kinase in integrin signaling. Int J Biochem Cell Biol.

[22] Zhao X, Guan JL (2011). Focal adhesion kinase and its signaling pathways in cell migration and angiogenesis. Adv Drug Deliv Rev.

[23] Cance WG (2000). Immunohistochemical analyses of focal adhesion kinase expression in benign and malignant human breast and colon tissues: correlation with preinvasive and invasive phenotypes. Clin Cancer Res.

[24] Cance WG, Liu ET (1995). Protein kinases in human breast cancer. Breast Cancer Res Treat.

[25] Owens LV (1995). Overexpression of the focal adhesion kinase (p125FAK) in invasive human tumors. Cancer Res.

[26] Lark AL (2003). Overexpression of focal adhesion kinase in primary colorectal carcinomas and colorectal liver metastases: immunohistochemistry and real-time PCR analyses. Clin Cancer Res.

[27] Golubovskaya V (2013). Disruption of focal adhesion kinase and p53 interaction with small molecule compound R2 reactivated p53 and blocked tumor growth. BMC Cancer.

[28] Fruman DA, Rommel C (2014). PI3K and cancer: lessons, challenges and opportunities. Nat Rev Drug Discov.

[29] Woltmann A, et al.: Systematic pathway enrichment analysis of a genome-wide association study on breast cancer survival reveals an influence of genes involved in cell adhesion and calcium signaling on the patients’ clinical outcome. PLoS One 2014, 9(6): doi:10.1371/journal.pone.0098229. 10.1371/journal.pone.0098229 PMC4041745 24886783

[30] Yang H, Zhang Q, He J, Lu W (2010). Regulation of calcium signaling in lung cancer. J Thorac Dis.

[31] Bailey C, Kelly P, Casey PJ (2009). Activation of Rap1 promotes prostate cancer metastasis. Cancer Res.

[32] Lu P, Weaver VM, Werb Z (2012). The extracellular matrix: A dynamic niche in cancer progression. J Cell Biol.

[33] Ardekani GS (2012). The prognostic value of *BRAF* mutation in colorectal cancer and melanoma: a systematic review and meta-analysis. PLoS One.

[34] Ho GY (2014). Circulating soluble cytokine receptors and colorectal cancer risk. Cancer Epidemiol Biomarkers Prev.

[35] Krupp M. et al.: The functional cancer map: A systems-level synopsis of genetic deregulation in cancer. BMC Medical Genomics 2011, 4(53). http://www.biomedcentral.com/1755-8794/4/53. 10.1186/1755-8794-4-53 PMC3148554 21718500

[36] Muzaffer MA (2013). Juvenile systemic lupus erythematosus and glioblastoma: a case report and literature review. Journal of King Abdulaziz University - Medical Sciences.

[37] Kulbe H (2004). The chemokine network in cancer - much more than directing cell movement. Int J Dev Biol.

[38] Van Dyke AL (2013). Cytokine and cytokine receptor single-nucleotide polymorphisms predict risk for non–small cell lung cancer among women. Cancer Epidemiol Biomarkers Prev.

[39] Spano JP (2004). Chemokine receptor CXCR4 and early-stage non-small cell lung cancer: pattern of expression and correlation with outcome. Ann Oncol.

[40] Banumathy G, Cairns P (2010). Signaling pathways in renal cell carcinoma. Cancer Biol Ther.

[41] Tang PA, Heng DY (2013). Programmed death 1 pathway inhibition in metastatic renal cell cancer and prostate cancer. Curr Oncol Re.

[42] Spurdle AB (2011). Genome-wide association study identifies a common variant associated with risk of endometrial cancer. Nat Genet.

[43] Baselga J (2011). Targeting the phosphoinositide-3 (PI3) kinase pathway in breast cancer. Oncologist.

[44] METABRIC Data for Use in Independent Research: https://www.synapse.org/#! Synapse:syn1688369.

