# Supplementary material for: Pan-cancer analysis of TCGA data reveals notable signaling pathways
Source: BMC Cancer. 2015 Jul 14;15:516. doi: 10.1186/s12885-015-1484-6 (PMC4501083; doi:10.1186/s12885-015-1484-6)
Supplement: Additional file 1: — These 11 tables show all pathways found to be significant (p-value < 0.05) in each of the analyses. Table S1. The pathways found to be significant in the pan-cancer analysis. Table S2. The pathways found to be significant in the breast cancer analysis. The far right column contains an entry if the pathway was found to be significant in the pan-cancer analysis. The entry is “H” if it was one of the highly significant pathways. Otherwise, it is “S”. Table S3. The pathways found to be significant in colon adenocarcinoma analysis. The far right column contains an entry if the pathway was found to be significant in the pan-cancer analysis. The entry is “H” if it was one of the highly significant pathways. Otherwise, it is “S”. Table S4. The pathways found to be significant in the glioblastoma analysis. The far right column contains an entry if the pathway was found to be significant in the pan-cancer analysis. The entry is “H” if it was one of the highly significant pathways. Otherwise, it is “S”. Table S5. The pathways found to be significant in the Kidney Renal Papillary Cell Carcinoma analysis. The far right column contains an entry if the pathway was found to be significant in the pan-cancer analysis. The entry is “H” if it was one of the highly significant pathways. Otherwise, it is “S”. Table S6. The pathways found to be significant in the Low Grade Glioma analysis. The far right column contains an entry if the pathway was found to be significant in the pan-cancer analysis. The entry is “H” if it was one of the highly significant pathways. Otherwise, it is “S”. Table S7. The pathways found to be significant in the Lung Adenocarcinoma analysis. The far right column contains an entry if the pathway was found to be significant in the pan-cancer analysis. The entry is “H” if it was one of the highly significant pathways. Otherwise, it is “S”. Table S8. The pathways found to be significant in the lung squamous cell carcinoma analysis. The far right column contains an en [file 12885_2015_1484_MOESM1_ESM.docx]

Table S1. The pathways found to be significant in the pan-cancer analysis.

| **Rank** | **Pathway** | **p-value** | **FDR** | **Status** |
| --- | --- | --- | --- | --- |
| 1 | Focal adhesion | 5.99E-06 | 0.000789 | Activated |
| 2 | PI3K-Akt signaling pathway | 1.01E-05 | 0.000789 | Activated |
| 3 | Rap1 signaling pathway | 3.71E-05 | 0.001939 | Activated |
| 4 | Calcium signaling pathway | 4.95E-05 | 0.001942 | Activated |
| 5 | Systemic lupus erythematosus | 0.001966 | 0.05302 | Activated |
| 6 | Pathways in cancer | 0.002026 | 0.05302 | Activated |
| 7 | FoxO signaling pathway | 0.003001 | 0.057233 | Activated |
| 8 | Melanoma | 0.003077 | 0.057233 | Activated |
| 9 | Adipocytokine signaling pathway | 0.003416 | 0.057233 | Activated |
| 10 | Prostate cancer | 0.003645 | 0.057233 | Activated |
| 11 | Gap junction | 0.004638 | 0.066193 | Activated |
| 12 | Cytokine-cytokine receptor interaction | 0.006201 | 0.081126 | Activated |
| 13 | Chemokine signaling pathway | 0.007867 | 0.095013 | Activated |
| 14 | Axon guidance | 0.011282 | 0.122407 | Activated |
| 15 | Acute myeloid leukemia | 0.011695 | 0.122407 | Activated |
| 16 | Adrenergic signaling in cardiomyocytes | 0.013894 | 0.136338 | Activated |
| 17 | Small cell lung cancer | 0.014839 | 0.137045 | Activated |
| 18 | ErbB signaling pathway | 0.017563 | 0.153186 | Activated |
| 19 | HTLV-I infection | 0.019726 | 0.162995 | Activated |
| 20 | Aldosterone-regulated sodium reabsorption | 0.024501 | 0.192329 | Activated |
| 21 | Viral carcinogenesis | 0.026088 | 0.195038 | Inhibited |
| 22 | Proteoglycans in cancer | 0.027349 | 0.195172 | Activated |
| 23 | Non-alcoholic fatty liver disease (NAFLD) | 0.031709 | 0.216449 | Activated |
| 24 | HIF-1 signaling pathway | 0.035074 | 0.229442 | Activated |
| 25 | Ras signaling pathway | 0.038097 | 0.239247 | Activated |
| 26 | Glioma | 0.041832 | 0.250529 | Activated |
| 27 | Amoebiasis | 0.045945 | 0.250529 | Activated |
| 28 | Cell cycle | 0.04614 | 0.250529 | Activated |
| 29 | Maturity onset diabetes of the young | 0.046754 | 0.250529 | Inhibited |
| 30 | RNA degradation | 0.048589 | 0.250529 | Inhibited |
| 32 | Fanconi anemia pathway | 0.050181 | 0.250529 | Inhibited |
| 33 | Complement and coagulation cascades | 0.051063 | 0.250529 | Activated |

Table S2. The pathways found to be significant in the breast cancer analysis. The far right column contains an entry if the pathway was found to be significant in the pan-cancer analysis. The entry is “H” if it was one of the highly significant pathways. Otherwise, it is “S”.

| **Rank** | **Pathway** | **p-value** | **FDR** | **Status** | **Pan_Cancer** |
| --- | --- | --- | --- | --- | --- |
| 1 | ECM-receptor interaction | 5.71E-05 | 0.008967 | Activated |  |
| 2 | Complement and coagulation cascades | 0.003855 | 0.218606 | Activated | S |
| 3 | Transcriptional misregulation in cancer | 0.006264 | 0.218606 | Activated |  |
| 4 | Systemic lupus erythematosus | 0.008005 | 0.218606 | Activated | S |
| 5 | Bile secretion | 0.008808 | 0.218606 | Inhibited |  |
| 6 | Endocrine and other factor-regulated calcium reabsorption | 0.009285 | 0.218606 | Inhibited |  |
| 7 | Pancreatic secretion | 0.010684 | 0.218606 | Inhibited |  |
| 8 | Fanconi anemia pathway | 0.011139 | 0.218606 | Activated | S |
| 9 | Adipocytokine signaling pathway | 0.016937 | 0.26641 | Inhibited | S |
| 10 | PPAR signaling pathway | 0.016969 | 0.26641 | Inhibited |  |
| 11 | Pathways in cancer | 0.02015 | 0.287598 | Inhibited | S |
| 12 | Alcoholism | 0.029112 | 0.380876 | Activated |  |
| 13 | Melanoma | 0.038254 | 0.383063 | Inhibited | S |
| 14 | ErbB signaling pathway | 0.038768 | 0.383063 | Inhibited | S |
| 15 | MAPK signaling pathway | 0.040044 | 0.383063 | Inhibited |  |
| 16 | Rap1 signaling pathway | 0.041062 | 0.383063 | Inhibited | H |
| 17 | Cell cycle | 0.043032 | 0.383063 | Inhibited | S |
| 18 | Protein processing in endoplasmic reticulum | 0.043918 | 0.383063 | Activated |  |
| 19 | Herpes simplex infection | 0.048081 | 0.397303 | Activated |  |

Table S3. The pathways found to be significant in colon adenocarcinoma analysis. The far right column contains an entry if the pathway was found to be significant in the pan-cancer analysis. The entry is “H” if it was one of the highly significant pathways. Otherwise, it is “S”.

| **Rank** | **Pathway** | **p-value** | **FDR** | **Status** | **Pan_Cancer** |
| --- | --- | --- | --- | --- | --- |
| 1 | Adrenergic signaling in cardiomyocytes | 3.35E-05 | 0.001709 | Inhibited | S |
| 3 | Melanoma | 3.68E-05 | 0.001709 | Inhibited | S |
| 4 | Focal adhesion | 4.73E-05 | 0.001709 | Inhibited | H |
| 5 | Cytokine-cytokine receptor interaction | 5.84E-05 | 0.001709 | Activated | S |
| 6 | Pathways in cancer | 6.21E-05 | 0.001709 | Inhibited | S |
| 7 | PI3K-Akt signaling pathway | 6.53E-05 | 0.001709 | Inhibited | H |
| 8 | Rap1 signaling pathway | 0.002919 | 0.065477 | Inhibited | H |
| 9 | Ras signaling pathway | 0.006911 | 0.135629 | Inhibited | S |
| 10 | Prostate cancer | 0.010696 | 0.186591 | Inhibited | S |
| 11 | Gap junction | 0.013127 | 0.20609 | Inhibited | S |
| 12 | Proteoglycans in cancer | 0.016654 | 0.234809 | Inhibited | S |
| 13 | Aldosterone-regulated sodium reabsorption | 0.017947 | 0.234809 | Inhibited | S |
| 14 | Glioma | 0.021402 | 0.256583 | Inhibited | S |
| 15 | Maturity onset diabetes of the young | 0.023444 | 0.256583 | Activated | S |
| 16 | ECM-receptor interaction | 0.024514 | 0.256583 | Inhibited | S |
| 17 | FoxO signaling pathway | 0.034098 | 0.334585 | Inhibited | S |
| 18 | Chemokine signaling pathway | 0.039736 | 0.366971 | Activated | S |
| 19 | Parkinson's disease | 0.042677 | 0.372239 | Activated |  |

Table S4. The pathways found to be significant in the glioblastoma analysis. The far right column contains an entry if the pathway was found to be significant in the pan-cancer analysis. The entry is “H” if it was one of the highly significant pathways. Otherwise, it is “S”.

| **Rank** | **Pathway** | **p-value** | **FDR** | **Status** | **Pan_Cancer** |
| --- | --- | --- | --- | --- | --- |
| 1 | Cytokine-cytokine receptor interaction | 5.12E-07 | 8.04E-05 | Inhibited | S |
| 2 | Complement and coagulation cascades | 1.33E-05 | 0.000798 | Inhibited | S |
| 3 | Systemic lupus erythematosus | 1.94E-05 | 0.000798 | Inhibited | S |
| 4 | PI3K-Akt signaling pathway | 2.31E-05 | 0.000798 | Inhibited | H |
| 5 | Chemokine signaling pathway | 2.54E-05 | 0.000798 | Inhibited | S |
| 6 | Vascular smooth muscle contraction | 0.003076 | 0.069809 | Inhibited |  |
| 7 | Autoimmune thyroid disease | 0.003113 | 0.069809 | Inhibited |  |
| 8 | Pathogenic Escherichia coli infection | 0.006026 | 0.111912 | Activated |  |
| 9 | Leukocyte transendothelial migration | 0.006415 | 0.111912 | Inhibited |  |
| 10 | Adrenergic signaling in cardiomyocytes | 0.011848 | 0.170169 | Inhibited | S |
| 11 | Allograft rejection | 0.012798 | 0.170169 | Inhibited |  |
| 12 | Legionellosis | 0.013205 | 0.170169 | Inhibited |  |
| 13 | Rap1 signaling pathway | 0.01409 | 0.170169 | Inhibited | H |
| 14 | Focal adhesion | 0.017319 | 0.172448 | Inhibited | H |
| 15 | Amoebiasis | 0.01779 | 0.172448 | Inhibited |  |
| 16 | Inflammatory bowel disease (IBD) | 0.017814 | 0.172448 | Inhibited |  |
| 17 | Type I diabetes mellitus | 0.018673 | 0.172448 | Inhibited |  |
| 18 | Pertussis | 0.020386 | 0.177808 | Inhibited |  |
| 19 | Mineral absorption | 0.028138 | 0.216844 | Inhibited |  |
| 20 | ECM-receptor interaction | 0.028683 | 0.216844 | Inhibited |  |
| 21 | Maturity onset diabetes of the young | 0.029005 | 0.216844 | Activated | S |
| 22 | Cell cycle | 0.032153 | 0.229456 | Inhibited | S |
| 23 | Graft-versus-host disease | 0.039804 | 0.251193 | Inhibited |  |
| 24 | Staphylococcus aureus infection | 0.041049 | 0.251193 | Inhibited |  |
| 25 | Fanconi anemia pathway | 0.041591 | 0.251193 | Activated |  |
| 26 | Serotonergic synapse | 0.041649 | 0.251193 | Activated |  |
| 27 | Aldosterone-regulated sodium reabsorption | 0.043199 | 0.251193 | Inhibited | S |
| 28 | Gap junction | 0.049576 | 0.277978 | Inhibited | S |

Table S5. The pathways found to be significant in the Kidney Renal Papillary Cell Carcinoma analysis. The far right column contains an entry if the pathway was found to be significant in the pan-cancer analysis. The entry is “H” if it was one of the highly significant pathways. Otherwise, it is “S”.

| **Rank** | | **Pathway** | **p-value** | **FDR** | **Status** | **Pan_Cancer** |
| --- | --- | --- | --- | --- | --- | --- |
| 1 | Rap1 signaling pathway | 3.30E-06 | 0.000518 | Inhibited | H |  |
| 2 | ECM-receptor interaction | 8.13E-06 | 0.000638 | Inhibited |  |  |
| 3 | Colorectal cancer | 2.79E-05 | 0.001459 | Inhibited |  |  |
| 4 | Focal adhesion | 8.66E-05 | 0.0034 | Inhibited | H |  |
| 5 | Insulin signaling pathway | 0.000557 | 0.015232 | Inhibited |  |  |
| 6 | Huntington's disease | 0.000582 | 0.015232 | Activated |  |  |
| 7 | Aldosterone-regulated sodium reabsorption | 0.000694 | 0.015562 | Inhibited | S |  |
| 8 | Axon guidance | 0.001441 | 0.028286 | Inhibited | S |  |
| 9 | Vascular smooth muscle contraction | 0.001971 | 0.032161 | Inhibited |  |  |
| 10 | Estrogen signaling pathway | 0.002048 | 0.032161 | Inhibited |  |  |
| 11 | PI3K-Akt signaling pathway | 0.003584 | 0.051155 | Inhibited | H |  |
| 12 | Wnt signaling pathway | 0.004048 | 0.05296 | Activated |  |  |
| 13 | Thyroid hormone signaling pathway | 0.004665 | 0.056336 | Inhibited |  |  |
| 14 | PPAR signaling pathway | 0.006543 | 0.073374 | Inhibited |  |  |
| 15 | Circadian entrainment | 0.008438 | 0.088317 | Inhibited |  |  |
| 16 | ErbB signaling pathway | 0.009171 | 0.089988 | Inhibited | S |  |
| 17 | Prolactin signaling pathway | 0.010915 | 0.098669 | Inhibited |  |  |
| 18 | Proteoglycans in cancer | 0.011312 | 0.098669 | Inhibited | S |  |
| 19 | Melanogenesis | 0.012244 | 0.098806 | Inhibited |  |  |
| 20 | Type II diabetes mellitus | 0.012936 | 0.098806 | Activated |  |  |
| 21 | Regulation of actin cytoskeleton | 0.013432 | 0.098806 | Inhibited |  |  |
| 22 | HIF-1 signaling pathway | 0.013845 | 0.098806 | Inhibited | S |  |
| 23 | Adipocytokine signaling pathway | 0.014991 | 0.102333 | Inhibited | S |  |
| 24 | Parkinson's disease | 0.017355 | 0.113534 | Activated |  |  |
| 25 | Acute myeloid leukemia | 0.018176 | 0.114144 | Inhibited | S |  |
| 26 | Non-alcoholic fatty liver disease (NAFLD) | 0.020627 | 0.124557 | Inhibited | S |  |
| 27 | GnRH signaling pathway | 0.024906 | 0.144822 | Inhibited |  |  |
| 28 | Chronic myeloid leukemia | 0.027859 | 0.148797 | Inhibited |  |  |
| 29 | Calcium signaling pathway | 0.027898 | 0.148797 | Inhibited | H |  |
| 30 | Endometrial cancer | 0.028433 | 0.148797 | Inhibited |  |  |
| 31 | Mineral absorption | 0.029842 | 0.151133 | Inhibited |  |  |
| 32 | Long-term depression | 0.031779 | 0.155917 | Inhibited |  |  |
| 33 | Alzheimer's disease | 0.034512 | 0.164191 | Activated |  |  |
| 34 | Pathways in cancer | 0.036337 | 0.167791 | Inhibited | S |  |
| 35 | Neurotrophin signaling pathway | 0.048024 | 0.215422 | Inhibited |  |  |
| 36 | Glioma | 0.050791 | 0.220247 | Inhibited | S |  |
| 37 | Salmonella infection | 0.052245 | 0.220247 | Inhibited |  |  |

Table S6. The pathways found to be significant in the Low Grade Glioma analysis. The far right column contains an entry if the pathway was found to be significant in the pan-cancer analysis. The entry is “H” if it was one of the highly significant pathways. Otherwise, it is “S”.

| **Rank** | **Pathway** | **p-value** | **FDR** | **Status** | **Pan_Cancer** |
| --- | --- | --- | --- | --- | --- |
| 1 | Focal adhesion | 4.94E-06 | 0.000674 | Inhibited | H |
| 2 | ECM-receptor interaction | 8.59E-06 | 0.000674 | Inhibited |  |
| 3 | Chemokine signaling pathway | 1.74E-05 | 0.00091 | Inhibited | S |
| 4 | Small cell lung cancer | 4.27E-05 | 0.001482 | Inhibited | S |
| 5 | Cytokine-cytokine receptor interaction | 4.72E-05 | 0.001482 | Inhibited | S |
| 6 | Retrograde endocannabinoid signaling | 0.000478 | 0.01252 | Activated |  |
| 7 | Circadian entrainment | 0.002716 | 0.056964 | Activated |  |
| 8 | Arrhythmogenic right ventricular cardiomyopathy (ARVC) | 0.002903 | 0.056964 | Inhibited |  |
| 9 | Cholinergic synapse | 0.005079 | 0.08337 | Activated |  |
| 10 | TNF signaling pathway | 0.00531 | 0.08337 | Inhibited |  |
| 11 | Mineral absorption | 0.007303 | 0.099086 | Inhibited |  |
| 12 | Long-term depression | 0.007573 | 0.099086 | Activated |  |
| 13 | PI3K-Akt signaling pathway | 0.009558 | 0.112746 | Inhibited | H |
| 14 | Estrogen signaling pathway | 0.010054 | 0.112746 | Activated |  |
| 15 | Phosphatidylinositol signaling system | 0.012223 | 0.124202 | Activated |  |
| 16 | HTLV-I infection | 0.01267 | 0.124202 | Inhibited | S |
| 17 | Tight junction | 0.013449 | 0.124202 | Inhibited |  |
| 18 | Morphine addiction | 0.028413 | 0.247825 | Activated |  |
| 19 | GABAergic synapse | 0.032095 | 0.26209 | Inhibited |  |
| 20 | Pathogenic Escherichia coli infection | 0.033387 | 0.26209 | Activated |  |
| 21 | Chagas disease (American trypanosomiasis) | 0.037645 | 0.281438 | Inhibited |  |
| 22 | Alcoholism | 0.047224 | 0.337008 | Inhibited |  |

Table S7. The pathways found to be significant in the Lung Adenocarcinoma analysis. The far right column contains an entry if the pathway was found to be significant in the pan-cancer analysis. The entry is “H” if it was one of the highly significant pathways. Otherwise, it is “S”.

| **Rank** | **Pathway** | **p-vlaue** | **FDR** | **Status** | **Pan_Cancer** |
| --- | --- | --- | --- | --- | --- |
| 1 | Chemokine signaling pathway | 1.82E-08 | 2.86E-06 | Activated | S |
| 2 | Cytokine-cytokine receptor interaction | 1.51E-05 | 0.001187 | Activated | S |
| 3 | Systemic lupus erythematosus | 0.000108 | 0.005654 | Activated | S |
| 4 | Rheumatoid arthritis | 0.00068 | 0.021445 | Activated |  |
| 5 | Viral carcinogenesis | 0.000683 | 0.021445 | Activated | S |
| 6 | Endocrine and other factor-regulated calcium reabsorption | 0.001515 | 0.039644 | Inhibited |  |
| 7 | Adrenergic signaling in cardiomyocytes | 0.002631 | 0.059016 | Inhibited | S |
| 8 | Focal adhesion | 0.004284 | 0.084074 | Inhibited | H |
| 9 | Tuberculosis | 0.00609 | 0.106235 | Activated |  |
| 10 | Prion diseases | 0.008503 | 0.121611 | Inhibited |  |
| 11 | Phosphatidylinositol signaling system | 0.008828 | 0.121611 | Activated |  |
| 12 | Adipocytokine signaling pathway | 0.009295 | 0.121611 | Inhibited |  |
| 13 | Alcoholism | 0.010633 | 0.128411 | Inhibited |  |
| 14 | Inflammatory bowel disease (IBD) | 0.012728 | 0.141185 | Activated |  |
| 15 | PPAR signaling pathway | 0.013832 | 0.141185 | Inhibited |  |
| 16 | Complement and coagulation cascades | 0.01467 | 0.141185 | Inhibited | S |
| 17 | Pathways in cancer | 0.015288 | 0.141185 | Inhibited | S |
| 18 | mTOR signaling pathway | 0.017379 | 0.147378 | Inhibited |  |
| 19 | Aldosterone-regulated sodium reabsorption | 0.0198 | 0.147378 | Inhibited | S |
| 20 | Melanoma | 0.020251 | 0.147378 | Inhibited | S |
| 21 | Maturity onset diabetes of the young | 0.020612 | 0.147378 | Activated | S |
| 22 | Natural killer cell mediated cytotoxicity | 0.020652 | 0.147378 | Activated |  |
| 23 | Salivary secretion | 0.022214 | 0.151637 | Inhibited |  |
| 24 | Antigen processing and presentation | 0.024633 | 0.161138 | Activated |  |
| 25 | Insulin signaling pathway | 0.033688 | 0.194236 | Inhibited |  |
| 26 | Chagas disease (American trypanosomiasis) | 0.033794 | 0.194236 | Activated |  |
| 27 | Glioma | 0.033936 | 0.194236 | Inhibited | S |
| 28 | Proteoglycans in cancer | 0.034641 | 0.194236 | Inhibited | S |
| 29 | Circadian entrainment | 0.036135 | 0.195628 | Inhibited |  |
| 30 | Pancreatic secretion | 0.038745 | 0.202765 | Activated |  |
| 31 | Oocyte meiosis | 0.040971 | 0.207497 | Activated |  |
| 32 | NF-kappa B signaling pathway | 0.043679 | 0.214301 | Activated |  |
| 33 | Apoptosis | 0.052982 | 0.252066 | Activated |  |

Table S8. The pathways found to be significant in the lung squamous cell carcinoma analysis. The far right column contains an entry if the pathway was found to be significant in the pan-cancer analysis. The entry is “H” if it was one of the highly significant pathways. Otherwise, it is “S”.

| **Rank** | **Pathway** | **p-vlaue** | **FDR** | **Status** | **Pan-Cancer** |
| --- | --- | --- | --- | --- | --- |
| 1 | Chemokine signaling pathway | 1.43E-05 | 0.002204 | Activated | S |
| 2 | Cytokine-cytokine receptor interaction | 4.14E-05 | 0.002204 | Activated | S |
| 3 | Endocrine and other factor-regulated calcium reabsorption | 4.21E-05 | 0.002204 | Inhibited |  |
| 4 | Amoebiasis | 0.005649 | 0.221723 | Inhibited | S |
| 5 | Tuberculosis | 0.014236 | 0.384387 | Activated |  |
| 6 | Rheumatoid arthritis | 0.024003 | 0.384387 | Activated |  |
| 7 | Systemic lupus erythematosus | 0.024563 | 0.384387 | Activated | S |
| 8 | Melanoma | 0.025304 | 0.384387 | Inhibited | S |
| 9 | Adipocytokine signaling pathway | 0.026468 | 0.384387 | Inhibited | S |
| 10 | Basal cell carcinoma | 0.029849 | 0.384387 | Activated |  |
| 11 | PI3K-Akt signaling pathway | 0.030259 | 0.384387 | Inhibited | H |
| 12 | Cell cycle | 0.031812 | 0.384387 | Inhibited | S |
| 13 | Rap1 signaling pathway | 0.035539 | 0.384387 | Inhibited | H |
| 14 | Inflammatory bowel disease (IBD) | 0.038635 | 0.384387 | Activated |  |
| 15 | Pancreatic secretion | 0.038725 | 0.384387 | Activated |  |
| 16 | Aldosterone-regulated sodium reabsorption | 0.039173 | 0.384387 | Inhibited | S |

Table S9. The pathways found to be significant in the ovarian cancer analysis. The far right column contains an entry if the pathway was found to be significant in the pan-cancer analysis. The entry is “H” if it was one of the highly significant pathways. Otherwise, it is “S”.

| **Rank** | **Pathway** | **p-value** | **FDR** | **Status** | **Pan_Cancer** |
| --- | --- | --- | --- | --- | --- |
| 1 | Rap1 signaling pathway | 4.02E-05 | 0.002785 | Inhibited | H |
| 2 | PI3K-Akt signaling pathway | 5.03E-05 | 0.002785 | Inhibited | H |
| 3 | Calcium signaling pathway | 5.32E-05 | 0.002785 | Inhibited | H |
| 4 | Focal adhesion | 0.000366 | 0.014354 | Inhibited | H |
| 5 | Glioma | 0.001827 | 0.057366 | Inhibited | S |
| 6 | ErbB signaling pathway | 0.005863 | 0.153412 | Inhibited | S |
| 7 | Adrenergic signaling in cardiomyocytes | 0.011648 | 0.26125 | Inhibited | S |
| 8 | Ras signaling pathway | 0.013458 | 0.264108 | Inhibited | S |
| 9 | Melanoma | 0.015183 | 0.264852 | Inhibited | S |
| 10 | PPAR signaling pathway | 0.019377 | 0.304217 | Inhibited |  |
| 11 | Gap junction | 0.022086 | 0.315222 | Inhibited | S |
| 12 | Pancreatic cancer | 0.029588 | 0.384379 | Inhibited |  |
| 13 | Chemokine signaling pathway | 0.031828 | 0.384379 | Inhibited | S |
| 14 | Endocrine and other factor-regulated calcium reabsorption | 0.045839 | 0.46715 | Inhibited |  |
| 15 | Cell cycle | 0.046637 | 0.46715 | Inhibited | S |
| 16 | Alzheimer's disease | 0.049006 | 0.46715 | Inhibited |  |
| 17 | FoxO signaling pathway | 0.050583 | 0.46715 | Inhibited | S |

Table S10. The pathways found to be significant in the rectum adenocarcinoma analysis. The far right column contains an entry if the pathway was found to be significant in the pan-cancer analysis. The entry is “H” if it was one of the highly significant pathways. Otherwise, it is “S”.

| **Rank** | **Pathway** | **p-value** | **FDR** | **Status** | **Pan-Cancer** |
| --- | --- | --- | --- | --- | --- |
| 1 | Focal adhesion | 3.63E-06 | 0.000342 | Inhibited | H |
| 2 | Rap1 signaling pathway | 4.36E-06 | 0.000342 | Inhibited | H |
| 3 | Ras signaling pathway | 1.32E-05 | 0.000689 | Inhibited | S |
| 4 | PI3K-Akt signaling pathway | 4.96E-05 | 0.001727 | Inhibited | H |
| 5 | Prostate cancer | 5.50E-05 | 0.001727 | Inhibited | S |
| 6 | Melanoma | 0.001514 | 0.039609 | Inhibited | S |
| 7 | FoxO signaling pathway | 0.003039 | 0.068167 | Inhibited | S |
| 8 | Pathways in cancer | 0.004142 | 0.081285 | Inhibited | S |
| 9 | ECM-receptor interaction | 0.006009 | 0.104827 | Inhibited |  |
| 10 | Proteoglycans in cancer | 0.007419 | 0.116472 | Inhibited | S |
| 11 | Glioma | 0.009139 | 0.130437 | Inhibited | S |
| 12 | Adrenergic signaling in cardiomyocytes | 0.011219 | 0.146783 | Inhibited | S |
| 13 | Gap junction | 0.013854 | 0.153075 | Inhibited | S |
| 14 | Leukocyte transendothelial migration | 0.014024 | 0.153075 | Inhibited |  |
| 15 | Aldosterone-regulated sodium reabsorption | 0.014625 | 0.153075 | Inhibited | S |
| 16 | Maturity onset diabetes of the young | 0.015931 | 0.156319 | Activated | S |
| 17 | p53 signaling pathway | 0.020686 | 0.191045 | Activated |  |
| 18 | HIF-1 signaling pathway | 0.026553 | 0.231603 | Inhibited | S |
| 19 | Calcium signaling pathway | 0.03085 | 0.254922 | Inhibited | H |
| 20 | Adipocytokine signaling pathway | 0.038611 | 0.286606 | Inhibited | S |
| 21 | Malaria | 0.038717 | 0.286606 | Inhibited |  |
| 22 | TGF-beta signaling pathway | 0.040161 | 0.286606 | Inhibited |  |
| 23 | Fanconi anemia pathway | 0.043967 | 0.300121 | Activated | S |
| 24 | Sulfur relay system | 0.048262 | 0.311299 | Activated |  |
| 25 | MicroRNAs in cancer | 0.04957 | 0.311299 | Inhibited |  |
| 26 | Mineral absorption | 0.052796 | 0.318807 | Activated |  |

Table S11. The pathways found to be significant in the uterine corpus endometrioid carcinoma analysis. The far right column contains an entry if the pathway was found to be significant in the pan-cancer analysis. The entry is “H” if it was one of the highly significant pathways. Otherwise, it is “S”.

| **Rank** | **Pathway** | **p-value** | **FDR** | **Status** | **Pan_Cancer** |
| --- | --- | --- | --- | --- | --- |
| 1 | Focal adhesion | 7.50E-07 | 0.000118 | Inhibited | H |
| 2 | Maturity onset diabetes of the young | 4.69E-05 | 0.003144 | Activated | S |
| 3 | Calcium signaling pathway | 6.01E-05 | 0.003144 | Inhibited | H |
| 4 | Rap1 signaling pathway | 0.005318 | 0.208728 | Inhibited | H |
| 5 | Chemokine signaling pathway | 0.007682 | 0.241209 | Inhibited | S |
| 6 | ErbB signaling pathway | 0.010835 | 0.28351 | Inhibited | S |
| 7 | Ras signaling pathway | 0.015805 | 0.33137 | Inhibited | S |
| 8 | Olfactory transduction | 0.020427 | 0.33137 | Activated |  |
| 9 | Neurotrophin signaling pathway | 0.021568 | 0.33137 | Inhibited |  |
| 10 | Glioma | 0.023477 | 0.33137 | Inhibited | S |
| 11 | Cytokine-cytokine receptor interaction | 0.025094 | 0.33137 | Inhibited | S |
| 12 | Fanconi anemia pathway | 0.027302 | 0.33137 | Activated | S |
| 13 | PPAR signaling pathway | 0.027828 | 0.33137 | Inhibited |  |
| 14 | Adrenergic signaling in cardiomyocytes | 0.029898 | 0.33137 | Inhibited | S |
| 15 | Adipocytokine signaling pathway | 0.03166 | 0.33137 | Inhibited | S |
| 16 | HIF-1 signaling pathway | 0.034141 | 0.335006 | Inhibited | S |
